# *GhBRX.1*, *GhBRX.2*, and *GhBRX4.3* improve resistance to salt and cold stress in upland cotton

**DOI:** 10.3389/fpls.2024.1353365

**Published:** 2024-02-09

**Authors:** Wei Wei, Jisheng Ju, Xueli Zhang, Pingjie Ling, Jin Luo, Ying Li, Wenjuan Xu, Junji Su, Xianliang Zhang, Caixiang Wang

**Affiliations:** ^1^ State Key Laboratory of Aridland Crop Science, College of Life Science and Technology, Gansu Agricultural University, Lanzhou, China; ^2^ Center for Western Agricultural Research, Chinese Academy of Agricultural Sciences (CAAS), Changji, China; ^3^ Institute of Cotton Research, State Key Laboratory of Cotton Biology, Chinese Academy of Agricultural Sciences (CAAS), Anyang, China

**Keywords:** BREVIS RADIX, salt stress, cold stress, virus-induced gene silencing (VIGS), upland cotton

## Abstract

**Introduction:**

Abiotic stress during growth readily reduces cotton crop yield. The different survival tactics of plants include the activation of numerous stress response genes, such as *BREVIS RADIX* (*BRX*).

**Methods:**

In this study, the *BRX* gene family of upland cotton was identified and analyzed by bioinformatics method, three salt-tolerant and cold-resistant *GhBRX* genes were screened. The expression of *GhBRX.1*, *GhBRX.2* and *GhBRXL4.3* in upland cotton was silenced by virus-induced gene silencing (VIGS) technique. The physiological and biochemical indexes of plants and the expression of related stress-response genes were detected before and after gene silencing. The effects of *GhBRX.1*, *GhBRX.2* and *GhBRXL4.3* on salt and cold resistance of upland cotton were further verified.

**Results and discussion:**

We discovered 12, 6, and 6 *BRX* genes in *Gossypium hirsutum*, *Gossypium raimondii* and *Gossypium arboreum*, respectively. Chromosomal localization indicated that the retention and loss of *GhBRX* genes on homologous chromosomes did not have a clear preference for the subgenomes. Collinearity analysis suggested that segmental duplications were the main force for *BRX* gene amplification. The upland cotton genes *GhBRX.1*, *GhBRX.2* and *GhBRXL4.3* are highly expressed in roots, and *GhBRXL4.3* is also strongly expressed in the pistil. Transcriptome data and qRT‒PCR validation showed that abiotic stress strongly induced *GhBRX.1*, *GhBRX.2* and *GhBRXL4.3*. Under salt stress and low-temperature stress conditions, the activities of superoxide dismutase (SOD), peroxidase (POD) and catalase (CAT) and the content of soluble sugar and chlorophyll decreased in *GhBRX.1-*, *GhBRX.2-* and *GhBRXL4.3*-silenced cotton plants compared with those in the control (TRV: 00). Moreover, *GhBRX.1*-, *GhBRX.2*- and *GhBRXL4.3*-silenced cotton plants exhibited greater malondialdehyde (MDA) levels than did the control plants. Moreover, the expression of stress marker genes (*GhSOS1*, *GhSOS2*, *GhNHX1*, *GhCIPK6*, *GhBIN2*, *GhSnRK2.6*, *GhHDT4D*, *GhCBF1* and *GhPP2C*) decreased significantly in the three target genes of silenced plants following exposure to stress. These results imply that the *GhBRX.1*, *GhBRX.2* and *GhBRXL4.3* genes may be regulators of salt stress and low-temperature stress responses in upland cotton.

## 1 Introduction

Cotton is an annual herbaceous plant of the Malvaceae family that is not only used to produce natural textile fibers but is also one of the major cash crops and a significant source of protein and seed oil worldwide (John and Crow, 1992). Despite the economic importance of cotton, various environmental factors, including both biotic and abiotic stresses, pose a great threat to cotton production (Potters et al., 2007). Due to global climate change, drought, salinity, extreme temperature, waterlogging, heavy metals, hypoxia and other major abiotic stresses will impede the development and growth of plants and affect crop yield and quality and sustainable agricultural development (Hasanuzzaman et al., 2020). To increase their chances of survival, plants have developed numerous defense strategies and mechanisms to handle a range of challenging circumstances (Westerheide et al., 2012; Van Zelm et al., 2020; Nian et al., 2021). In plant defense mechanisms, many stress-response genes help plants tolerate the adverse effects of various stressors by regulating their transcriptome levels.

The highly conserved *BREVIS RADIX* (*BRX*) family of plant-specific genes is found in all higher plants for which data are known but not in animals or single-celled organisms (Mouchel et al., 2004). For the first time, *BRX* was isolated from *Arabidopsis thaliana* UK-1 plants with a short root phenotype using map-based cloning (Mouchel et al., 2004). There are five *BRX* genes in *Arabidopsis*: *BRX*, *BRXL1* to *BRXL4*. Although the sequences of *BRX* are highly conserved, the functions of the five genes in the *Arabidopsis BRX* family are largely nonredundant (Briggs et al., 2006). BRX proteins have four highly conserved domains, two of which are short N-terminal domains and two of which are BRX domains (Briggs et al., 2006; Beuchat et al., 2010a; Liu et al., 2010). These include a 9–10 amino acid region at the N-terminus that is thought to contain palmitoylation signals and is crucial for the membrane localization of *BRX* (Rowe et al., 2019). The adjacent domains are a 25-amino acid N-terminal domain with a KDMA motif and two BRX domains with 55 amino acid extensions consisting of tandem repeats (Koh et al., 2021). The BRX domain may represent a new protein–protein interaction domain, which is the first indication of the biological function of the BRXL protein (Briggs et al., 2006). Adding one BRX domain to the corresponding BRX^N140^ fragment partially restored functionality (Briggs et al., 2006). However, the addition of two BRX domains to the corresponding BRX^N140^ fragment, as with full-length *BRX*, significantly alleviated the *brx* root growth phenotype and elicited hypocotyl function to acquire the phenotype (Briggs et al., 2006; Scacchi et al., 2009). The conserved N-terminal domain of BRX family proteins may play only secondary functional roles (Briggs et al., 2006). However, according to *Ka*/*Ks* analysis, the diversity of *BRX* family genes may be caused by the variable N-terminal region, which could be the cause of the nonredundant functions of most *AtBRX* family genes (Briggs et al., 2006; Beuchat et al., 2010a).

*BRX* is a growth regulator needed for root growth that regulates cell proliferation and elongation in root growth areas (Mouchel et al., 2006). Auxin substantially stimulates *BRX* expression, while brassinosteroid (BR) marginally inhibits expression (Mouchel et al., 2006). This finding suggests that *BRX* forms a feedback loop between BR and auxin, which maintains brassinosteroid thresholds and controls the root response to auxin, while auxin completes the cycle by controlling *BRX* expression (Mouchel et al., 2006). The *BRX* functional allele (*brx-2*) is highly sensitive to ABA-mediated root growth inhibition, and it has also been shown to be insensitive to cytokinin-induced lateral root initiation inhibition, indicating crosstalk between BR and cytokinin (Bari and Jones, 2009; Scacchi et al., 2009). Therefore, *BRX* could be a key node in the interconnection of auxin, BR, ABA, and cytokinin signaling during root development. (John and Crow, 1992; Scacchi et al., 2009). The BRX protein is associated with the plasma membrane but is translocated to the nucleus after auxin treatment to regulate gene expression (Scacchi et al., 2009). The *BRX* gene family has been studied and identified in a variety of plant species (Li et al., 2009; Liu et al., 2010; Zhang Y. et al., 2021; Tiwari et al., 2023). *BRX* is involved in the longitudinal and radial expansion of hypocotyls and roots, the development of embryos and leaves, and the asymmetric division of stomatal lineage cells in *Arabidopsis*, and unlike their partial or nonredundant roles in roots, *BRX* genes play redundant roles in stomatal development (Rowe et al., 2019). The *brx* mutants also exhibited significant reductions in cotyledon and leaf growth, and deletion of the *BRX* functional allele (*brx-2*) resulted in a decrease in rosette area in comparison to that of Col-0, but the quantity of leaves remained the same (Rodrigues et al., 2009). In contrast, plants with functionally acquired *BRX* exhibit elongated hypocotyls and epicotyl leaves (Scacchi et al., 2009; Gill and Tuteja, 2010). In rice, compared with those of WT plants, *OsBRXL4*-overexpressing transgenic plants had significantly longer roots and greater sensitivity to auxin under normal growth conditions (Liu et al., 2010). These results suggest that *OsBRXL4* may regulate primary root growth via auxin signaling (Liu et al., 2010). The optimal tillering angle is essential for an ideal plant structure, and the molecular mechanism controlling the tillering angle in rice will improve our ability to rationally change the structure of rice plants, thereby increasing grain yield. *OsBRX* regulates auxin transport to control the tiller angle of rice plants. (Scacchi et al., 2009; Liu et al., 2010). Overexpression of three different *BrBRX* genes in *Brassica rapa* significantly increased the number of rosette leaves, decreased the rosette area and increased the petiole length in transgenic plants (Zhang Y. et al., 2021). *TaBRXL1* is generally expressed in all analyzed tissues except flag leaves, and the expression levels of *TaBRXL2*, *TaBRXL3* and *TaBRXL4* are significantly increased under auxin treatment, indicating that *TaBRX* family genes may contribute to functional diversity (Tiwari et al., 2023).

Studies have shown that the expression of *BRX*s is differentially induced by different types of abiotic stress (Liu et al., 2010; Tiwari et al., 2023). In rice, *OsBRXL1* and *OsBRXL4* respond to drought, salt and cold stress; *OsBRXL3* responds to drought and salt stress; *OsBRXL2* and *OsBRXL5* respond only to cold stress; and the expression of these five *OsBRXL* genes is upregulated under drought and salt stress and downregulated under low-temperature stress (Liu et al., 2010). *TaBRXL1* was found to be involved primarily in developmental processes, whereas *TaBRXL2* was highly regulated by development, hormones, and other abiotic stimuli (Tiwari et al., 2023). In addition to *TaBRXL2*, the other *TaBRX* genes were significantly downregulated under drought conditions in common wheat. Under osmotic stress (200 mM mannitol), *TaBRXL2*, *TaBRXL3* and *TaBRXL4* were upregulated (Tiwari et al., 2023). In summary, the *BRX* gene family plays an important role in enhancing plant tolerance to abiotic stress.

Cotton is a valuable economic crop that provides “oil, fiber, feed, and medicine.” Yield losses readily occur due to abiotic stress during the growth development process of cotton. Thus, it is important to screen and apply key genes in cotton that respond well to abiotic stress and improve cotton stress resistance through biological breeding. To date, the relationship between the *GhBRX* gene and abiotic stress has not been studied. The whole genomes of 12 *GhBRX* genes were discovered in this work. Its evolutionary model, physical and chemical properties, chromosomal location, gene structure, *cis*-acting elements and expression pattern were comprehensively analyzed. Using the virus-induced gene silencing (VIGS) approach, we further elucidated the biological function of the *GhBRX.1*, *GhBRX.2* and *GhBRXL4.3* genes in response to salt and cold stress.

## 2 Materials and methods

### 2.1 Identification of *BRX* genes

To identify cotton *BRX* gene families, we used CottonFGD (http://www.cottonfgd.org/) and *Arabidopsis thaliana* genome sequence data from TAIR (http://www.arabidopsis.org) for five AtBRX protein sequences from Pfam (https://pfam.xfam.org/), which was subsequently used to download the PF08381 hidden Markov model (HMM) version 3.0 (El-Gebali et al., 2019). Then, we used HMMER 3.0 software with default parameter settings (http://www.HMMER.org/) to obtain the *BRX* gene, for which the E value was <1e^-5^ (Zhu et al., 2017). We used Pfam (https://www.ebi.ac.uk/Tools/pfa/pfamscan/) and SMART (https://smart.embl.de/) to further evaluate the results of our genes for confirmation. Finally, we manually confirmed the identified *BRX* genes. The BRX protein sequence was predicted through ExPASy (https://us.expasy.org/tools/protparam.html) to predict the molecular weight (MW), theoretical isoelectric point (pI), etc. In addition, Wolfpsort (https://www.wolfpsort.hgc.jp/) was used to predict the subcellular localization of the cotton BRX protein.

### 2.2 Sequence alignment and phylogenetic analysis

The ClustalW program (version 2.0) was used to align the full-length amino acid sequences of the *BRX*-encoded *Gossypium hirsutum* (*Gh*), *Gossypium arboreum* (*Ga*), *Gossypium raimondii* (*Gr*), *Arabidopsis thaliana* (*At*), *Brassica rapa* (*Br*), *Oryza sativa* (*Os*), and *Triticum aestivum* (*Ta*). The alignment was then manually modified in MEGA 7.0. Subsequently, we constructed a neighbor joining (NJ) tree with 1000 bootstrap repetitions using MEGA 7.0’s Poisson substitution model with default parameters (Kumar et al., 2016). The Interactive Tree of Life (iTOL) tool was utilized to enhance the visualization of the phylogenetic tree (http://itol.embl.de/).

### 2.3 Analysis of conserved gene structures and protein motifs

To find conserved protein motifs, we utilized the motif elicitation (multiple EM for motif elicitation) website (http://meme-suite.org/) (Bailey et al., 2009). A conservative motif map was generated using TBtools software (Chen et al., 2018). The upland cotton CDS and genome sequence and NWK file from phylogenetic tree analysis were used to map gene structure through the Server (Gene Structure Display Server, GSDS) program (http://gsds.cbi.pku.edu.cn/).

### 2.4 Analysis of chromosomal positions and gene collinearity

GFF3 files extracted from the CottonFGD database were used to determine chromosome locations. The *GhBRX* gene was located on the chromosome using TBtools software (Chen et al., 2020). During collinear analysis, to compare the GhBRX protein sequences, the Basic Local Alignment Search Tool (BLAST) was utilized, and the cutoff E value was < 10^-5^. The MCScanX tool of the TBtools software was subsequently used to evaluate the above BLASTP results, extract collinear pairs of GhBRX family proteins, and construct a collinearity map of the *GhBRX* family using TBtools (Chen et al., 2020). *Ka*/*Ks* values of the *GhBRX* gene were determined by using TBtools (Chen et al., 2020). TBtools was subsequently used to construct interspecific collinearity maps of *G. hirsutum*, *G. raimondii* and *G. arboretum* (Chen et al., 2020).

### 2.5 *Cis*-regulatory element analysis

We obtained an upstream sequence of our genes spanning 2 kb from the translation start site from CottonFGD (https://cottonfgd.net/) to identify *cis*-regulatory regions in *BRX* genes. Then, we predicted the *cis*-regulatory elements in the promoter region of the *GhBRX* genes using the PlantCARE website (http://bioinformatics.psb.ugent.be/beg).

### 2.6 Expression pattern analysis

To verify the *GhBRX* gene expression profile in upland cotton organizations, RNA-seq data from Zhejiang University (ZJU) (http://cotton.zju.edu.cn/) were downloaded to determine the *GhBRX* gene organization and response to salt, drought, cold and heat stresses(Zhang et al., 2015). Heatmaps of 12 *GhBRX* genes were generated using TBtools.

### 2.7 Plant material, RNA extraction, and fluorescence quantitative PCR

Healthy plants of the new upland cotton variety XinshiK25 were selected and treated with 15% PEG (PEG-6000), 250 mmol/L NaCl, 12°C and 42°C, respectively. The leaves were removed every 3 h and treated for 24 h. RNA and reverse transcription cDNA were extracted using a kit produced by Tiangen Biochemical Technology (Beijing) Co., Ltd. *GhBRX* gene primers were constructed using NCBI Prime-BLAST (primer design tool, https://www.ncbi.nlm.nih.gov/tools/primer-blast/index.cgi?LINK_LOC=BlastHome). **Supplementary Table S1** shows the primers used. Real-time fluorescence quantitative PCR was performed (SYBR Green, FP209, Tiangen, China) according to the instructions for the thermal cycling process. AY305733 is an internal control gene that employs the 2^-ΔΔ^ *^C^*_T_ technique (Livak and Schmittgen, 2001). The comparative expression values of *GhActin* and *GhBRX*s were calculated, and the relative expression levels of three independent biological replicates and technical replicates were averaged.

### 2.8 Cotton vector creation and the VIGS technique

The coding sequences of the *GhBRX.1*, *GhBRX.2* and *GhBRXL4.3* genes were downloaded from CottonFGD, and specific primers (**Supplementary Table S2**) were designed. Using the online tool NCBI Prime-BLAST, specific primers for gene silencing and related stress response gene fluorescence were designed (**Supplementary Table S3**). Specific cDNA sequences of *GhBRX.1*, *GhBRX.2* and *GhBRXL4.3* were amplified, constructed into Figure vectors, and subsequently introduced into Agrobacterium strain GV3101. Then, the cotyledons of two 8-day-old XinshiK25 plants were grown at 25°C for 24 h in the dark and injected with the vector (Gao et al., 2013). RNA was collected from cotton leaves that had been silenced at the four-leaf stage, and RT–qPCR was used to assess the silencing efficacy. RT–qPCR was used to test the expression of nine stress-responsive genes, *GhSOS1*, *GhSOS2*, *GhNHX1*, *GhCIPK6*, *GhBIN2, GhSnRK2.6*, *GhHDT4D*, *GhCBF1* and *GhPP2C*, associated with salt stress and cold stress in control and silenced plants.

### 2.9 Physiological and biochemical parameters of the silenced and control plants under salt and cold stress conditions

The activities of peroxidase (POD), catalase (CAT), and superoxide dismutase (SOD), three antioxidant enzymes that are important to plants under abiotic stress conditions, were evaluated. SOD activity was determined by tracking the suppression of the photochemical reduction of nitroblue tetrazole (Giannopolitis and Ries, 1977). POD activity was evaluated using ortho-methoxyphenol (guaiacol) as a substrate (Castro et al., 2017). POD can oxidize guaiacol to o-4-methoxyphenol, which can be detected via spectrophotometry at 470 nm. CAT activity is determined by UV absorption (Beers and Sizer, 1952), and the malondialdehyde (MDA) content is a marker of lipid peroxidation (Liu et al., 2018). Chlorophyll was extracted with 95% ethanol and measured spectrophotometrically at 665 nm and 649 nm (Lichtenthaler and Wellburn, 1983).

## 3 Results

### 3.1 Identification of *BRX*s in cotton

The amino acid sequences of the BRX proteins found in *Arabidopsis* and rice were used as query sequences. As shown in **Supplementary Table S4**, the presence of 12, 6, and 6 *BRX* genes was confirmed in *G. hirsutum*, *G. raimondii* and *G. arboreum*. The number of *BRX* genes in allotetraploid cotton was twice as high as that in the two diploid cotton lines, suggesting that the *BRX* gene family experienced expansion during evolution in *Gossypium* spp. while maintaining their unique genetic makeup. The predicted protein sequences were used to calculate the number of amino acids, MW, and pI. The 24 BRX proteins that were found had amino acid ranges of 342–405, protein pIs that varied from 5.73 to 8.59, and MWs that varied from 38.58 to 45.59 kDa. Bioinformatics analysis revealed that 24 BRX proteins were predicted to locate in the nucleus. All features and chromosomal locations of the identified *BRX*s are shown in Additional file **Supplementary Table S4**.

### 3.2 Phylogenetic analysis of the *BRX* genes

**Figure 1:**
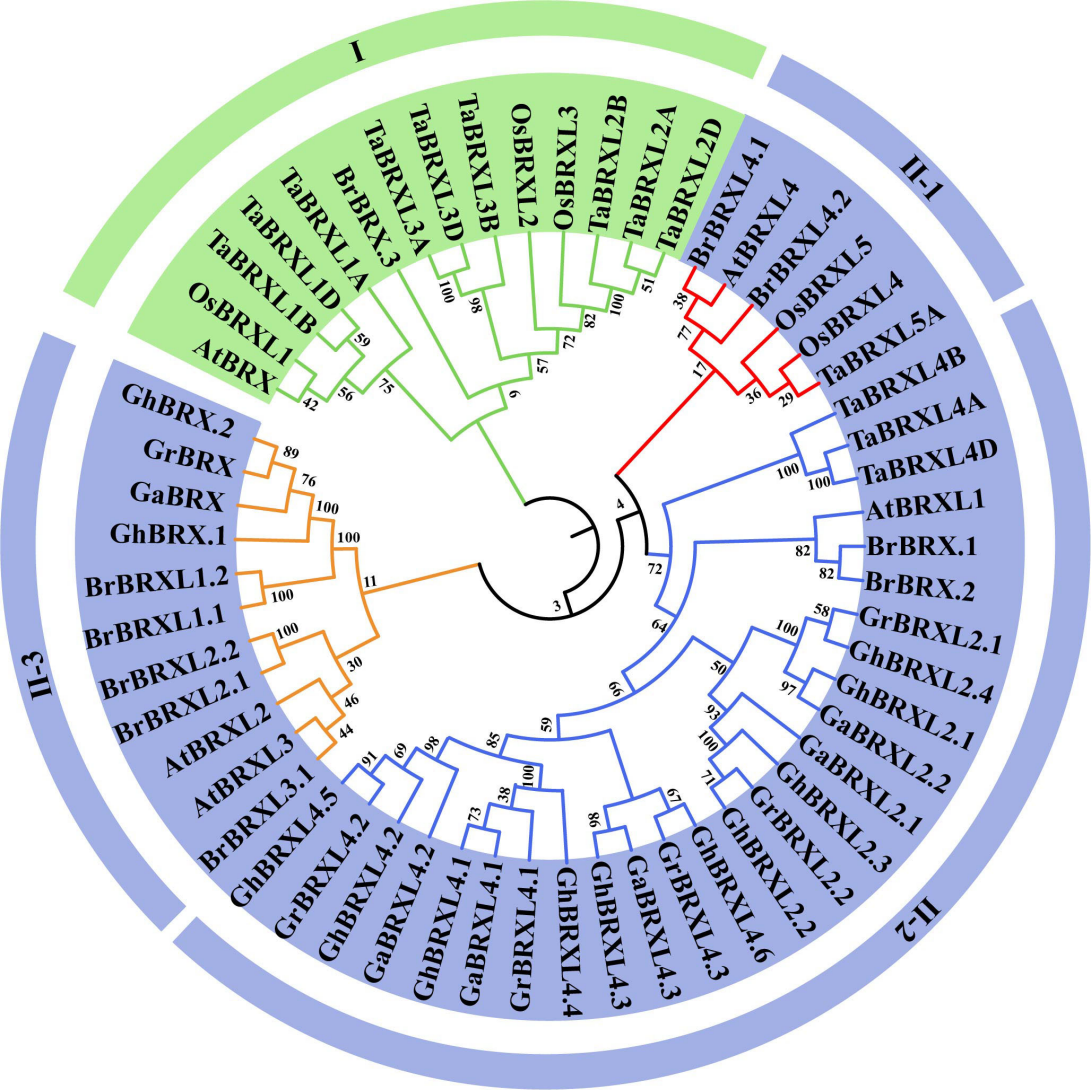
Phylogenetic relationships of BRX proteins in seven plant species. *Gossypium hirsutum (Gh), Gossypium raimondii (Gr), Gossypium arboreum (Ga), Brassica rapa (Br), Arabidopsis thaliana (At), Tariticum aestivum (Ta)*, and *Oryza sativa (Os)*. At each node, the bootstrap values were displayed.

Using 57 BRX protein sequences obtained from *G. hirsutum, G. raimondii, G. arboreum*, *B. rapa*, *A. thaliana*, *T. aestivum*, and *O. sativa*, we constructed a phylogenetic tree based on multiple alignment analyses using the neighbor–joining (NJ) method to examine the phylogenetic relationships among the *BRX* family genes (**Figure 1**). Based on bootstrap values (=1,000), the 57 BRX proteins were found to precluster into two main groups (Group I and Group II). Group I was defined by *AtBRX* (*At1G31880*) and had no *BRX* members from the three cotton species. Group II was divided into three subgroups: II-1, II-2 and II-3. All the *OsBRX* genes clustered in group I and subgroup II-1, and all three *Gossypium* spp. species *BRX* genes clustered in subgroups II-2 and II-3. As shown in **Figure 1**, mutually homologous *BRX* family genes clustered together, with one copy in diploid cotton species and two copies in heterotetraploid cotton species in almost every direct homologous group. The results of the clustering analysis provide additional evidence that the upland cotton species heterotetraploid is the product of an ancestral cross between *G. raimondii* and *G. arboreum*, two diploid cotton species, and doubles in number.

### 3.3 Structure and conserved motif analysis of *GhBRX* genes

We analyzed the structural features of *GhBRX* using the GSDS program and the exon and intron structures and conserved structural domains using the MEME tool. The *GhBRX* genes exhibited a comparatively high level of structural similarity according to the exon/intron structure analysis (**Supplementary Figure S1A**). All the *GhBRX* genes contained five exons. Most of the homologous genes had similar gene lengths, with *GhBRXL4.3* being the longest. The results suggest that exon structure is associated with phylogenetic relationships, further supporting the structural classification of the *GhBRX* gene family. Using MEME software, the functional regions of the GhBRX proteins were divided into five different motifs (**Supplementary Figure S1B**). Motifs 1, 2, 3 and 5 (BRX motifs) were widely distributed among all *GhBRX* family members, with only *GhBRX.1* and *GhBRX.2* without motif 4; these two genes also had the shortest lengths. The motif compositions of GhBRX proteins in a given branch were strikingly similar, indicating that these proteins may early every play comparable role.

### 3.4 Genetic replication and collinearity analysis of *GhBRX*

**Figure 2:**
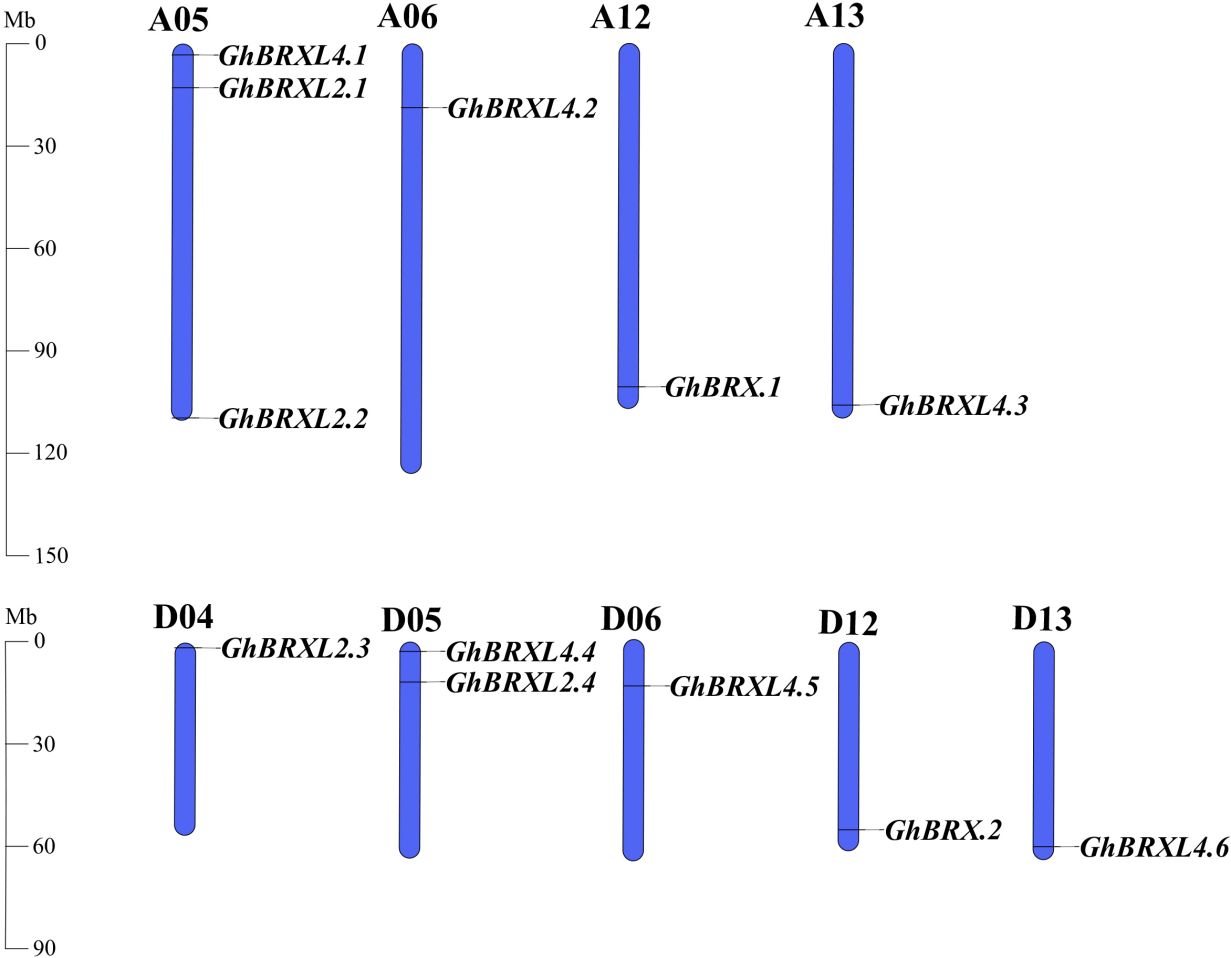
*GhBRX* locations on *Gossypium hirsutum* chromosomes. The blue bars represent the At and Dt subgenomes of *G. hirsutum*. The subgenomes’ matching gene names were showned on the right side of each chromosome.

**Figure 3:**
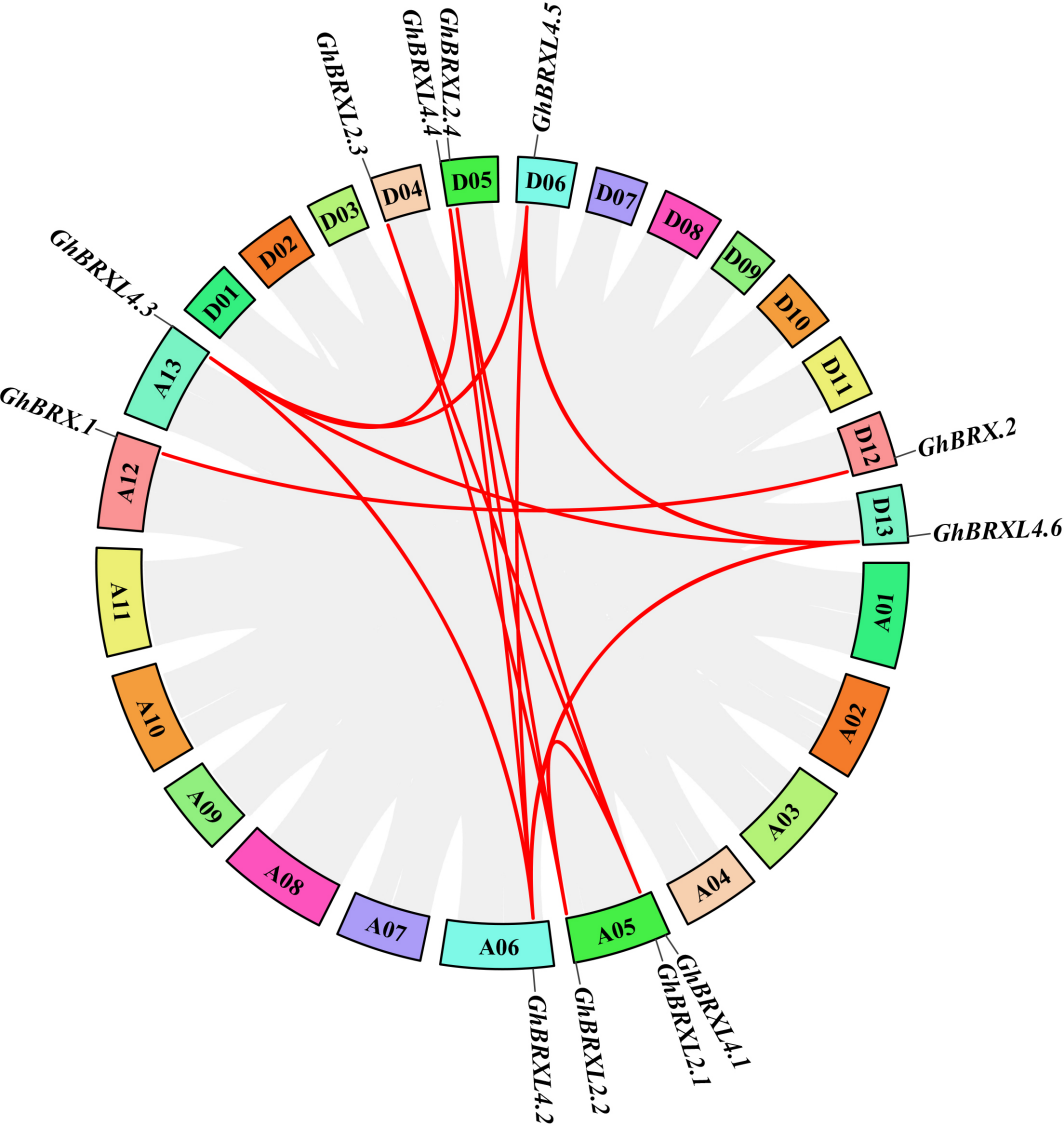
Duplication of *GhBRX* genes. Duplications of *GhBRX* genes on the chromosome of *G. hirsutum*; the red lines represented gene pairs of *GhBRX*. The rectangles represented chromosomes.

Gene duplication events in *G. hirsutum* were explored with TBtools to elucidate the amplification patterns and determine the homologous locus linkages of the *GhBRX* gene family members between the At and Dt subgenomes (**Figure 2**). One tandem duplicate gene was identified on chromosome A05. In addition, the *GhBRX* gene family contained 20 segmentally duplicated genes (**Figure 3**). These data suggest that segmental duplications are important in the evolution of *GhBRX* gene families, indicating the dominance of segmental repeats over tandem repeats in the evolution of the *GhBRX* gene family (**Supplementary Table S5**). Furthermore, to gain a deeper understanding of the homologous gene functions and evolutionary relationships of the *BRX* genes, the outcomes of the genome symbiosis study between upland cotton and two other species of cotton were examined (**Supplementary Figure S2**). In conclusion, the current findings suggest that *BRX* genes may undergo certain genomic rearrangements during polyploidy. The nonsynonymous substitution (*Ka*), synonymous substitution (*Ks*), and *Ka*/*Ks* ratio were estimated to better understand the evolutionary constraints regulating the functional divergence of the *GhBRX* gene family (**Supplementary Table S6**). The *Ka*/*Ks* ratio of all duplicate *GhBRX* gene pairs was less than 1, indicating that selective pressure may have been applied to the *GhBRX* family genes throughout their evolutionary history.

### 3.5 Analysis of *cis*-elements in the promoters of *GhBRX*s

**Figure 4:**
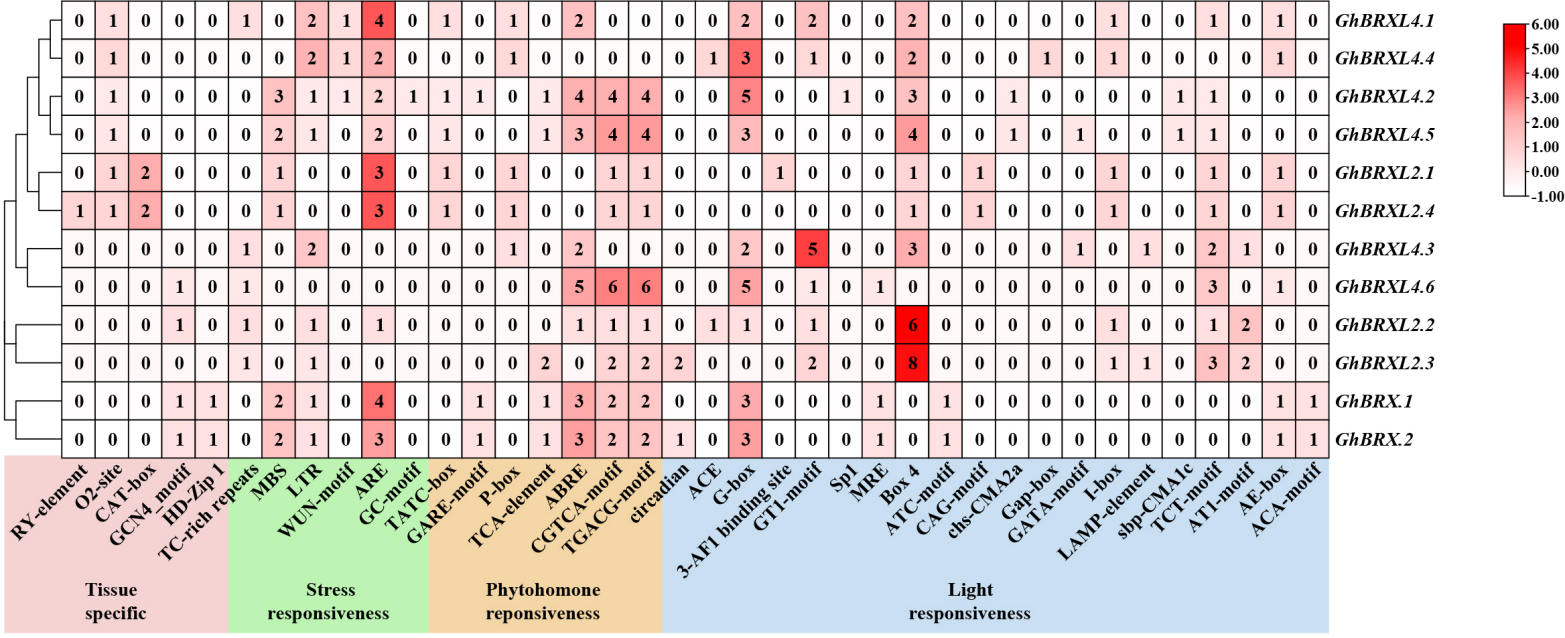
*Cis*-regulatory element prediction outcomes in the promoter regions of *GhBRX* gene family members. Shades and digits in the boxes denoted the number of *cis*-elements.

We examined and characterized the *cis*-acting region in the 2 kb promoter sequence of *GhBRX*s to further investigate the putative regulatory roles of th*e BRX* gene in response to abiotic stressors. We discovered 38 different types of *cis*-regulatory elements that are involved in tissue-specific expression, stress responses, phytohormone responses, and light responses (**Figure 4**). Five elements associated with tissue-specific expression were identified, namely, the RY element, O2 site, CAT box, GCN4 motif and HD-Zip 1. The *cis*-regulatory element associated with meristematic tissue expression (CAT-box) was only present in the promoter regions of the homologs *GhBRXL2.1* and *GhBRXL2.4*, and the seed-specific regulatory element (RY-element) is specific to *GhBRXL2.4*. Six elements associated with stress responsiveness were identified, and these *cis*-acting elements are involved in defense and stress responsiveness, drought, low temperature, anaerobic and wounding responses. Abiotic stress response elements, such as the drought response element (MBS) and low temperature response element (LTR), were present in variable amounts in the homologous genes *GhBRX.1*, *GhBRX.2*, *GhBRXL4.2* and *GhBRXL4.5*. The *cis*-acting regulatory elements necessary for anaerobic induction (ARE) were more abundant in most *GhBRX* genes. In addition, four hormone-related elements were identified, namely, the gibberellin response element (GARE motif-containing element, P-box and TATC motif-containing element), the abscisic acid response element (ABRE), the salicylic acid response element (TCA-element) and the methyl jasmonate (MeJA) response element (CGTCA motif-containing element and TGACG motif-containing element). Among them, ABRE and MeJA response elements were relatively abundant in most *GhBRX* genes. Light-responsive *cis*-elements, including Box 4, G-box, and TCT-motif, were present in all the *GhBRX* promoters. Among the light-responsive *cis*-acting regulatory elements, Box 4 and G-box were relatively more common. Taken together, these findings imply that *GhBRX* genes might be crucial for defense-related signaling, phytohormone responses, and abiotic stress responses.

### 3.6 Expression analysis of *BRX* genes in upland cotton

**Figure 5:**
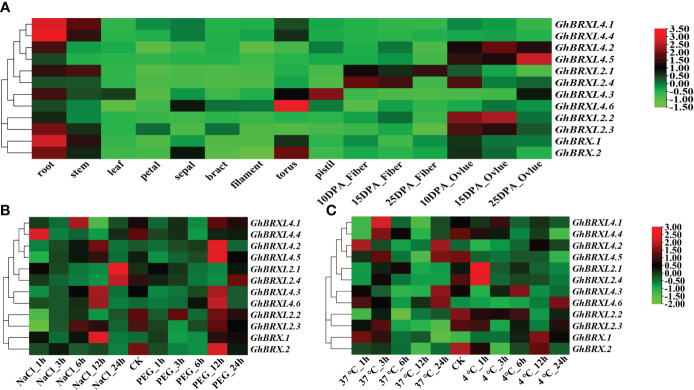
*GhBRX* gene expression patterns in distinct tissues and under four abiotic stimuli in upland cotton. **(A)** Expression patterns of 12 *GhBRX*s in various tissues. **(B)** Salt and PEG stress expression patterns of 12 *GhBRX*s. **(C)** Expression patterns of 12 *GhBRX*s under heat and cold stress.

To determine the purpose of the *GhBRX* gene, we analyzed the expression profile data in the cotton functional database. Tissue-specific expression analysis indicated that the *GhBRX.1*, *GhBRX.2*, *GhBRXL2.3*, *GhBRXL4.1* and *GhBRXL4.4* genes were expressed mainly in roots; *GhBRXL2.2*, *GhBRXL4.2*, and *GhBRXL4.5* were expressed mainly in ovules; and *GhBRXL2.4*, *GhBRXL4.3* and *GhBRXL4.6* were expressed highly in fibers, pistils, and flowers, respectively (**Figure 5A**). According to the expression analysis, the response of five genes, namely, *GhBRX.1*, *GhBRXL2.1*, *GhBRXL2.4*, *GhBRXL4.3* and *GhBRXL4.6*, to abiotic stress significantly increased under salt treatment (**Figure 5B**). Under PEG stress, *GhBRX.2*, *GhBRXL4.2*, *GhBRXL4.3*, *GhBRXL4.5* and *GhBRXL4.6* were also significantly upregulated (**Figure 5B**). Under heat stress treatment, four genes were upregulated, namely, *GhBRXL4.1*, *GhBRXL4.2*, *GhBRXL4.3* and *GhBRXL4.6* (**Figure 5C**). Under cold stress treatment, *GhBRX.1* and *GhBRXL4.6* were upregulated, while *GhBRXL2.1* and *GhBRXL2.4* were downregulated (**Figure 5C**).

### 3.7 Expression of *GhBRX* genes in response to abiotic stresses

**Figure 6:**
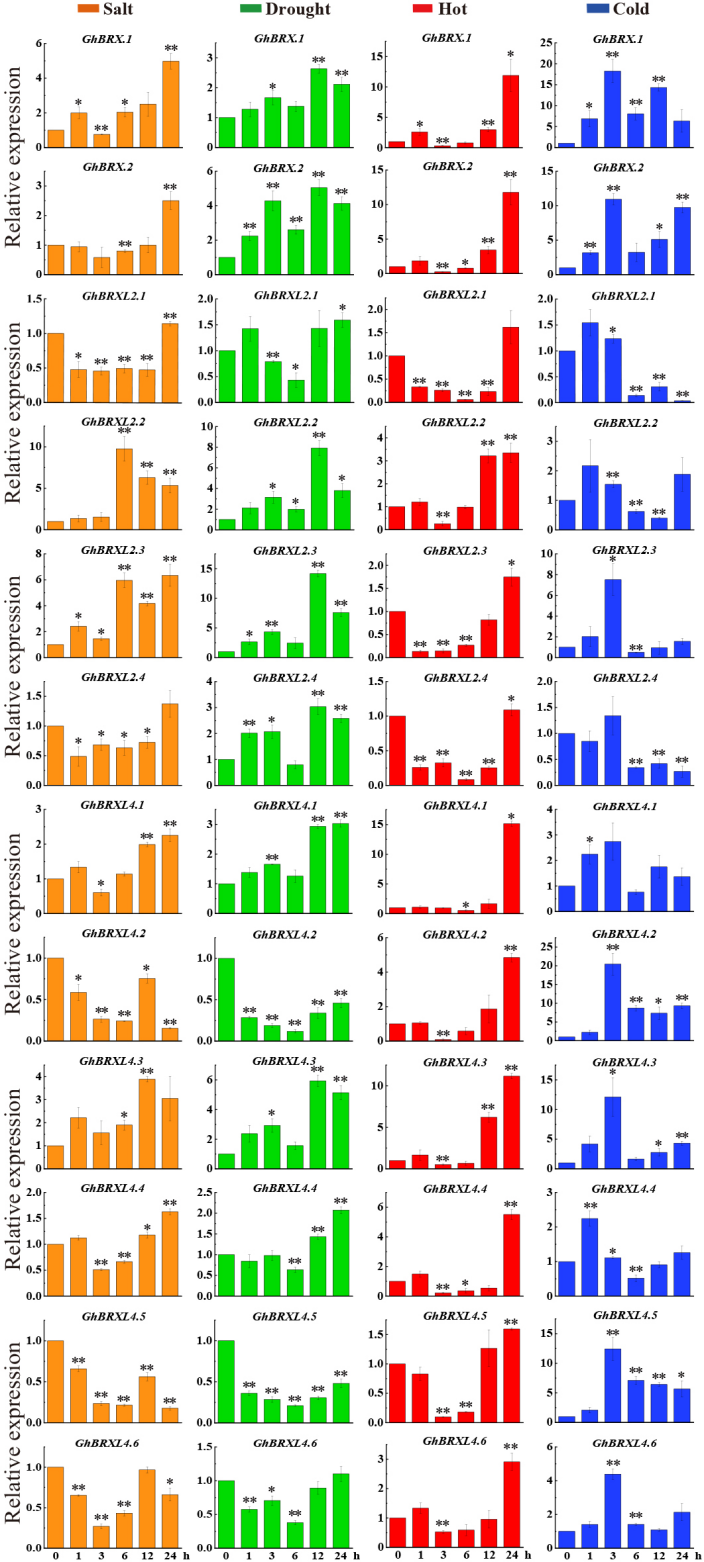
Relative *GhBRX*s expression levels in response to heat, cold, drought, and salt treatments. The standard deviations of the three biological replicates were represented by error bars. Orange denoted salt stress, green denoted drought stress, red denoted heat stress, and blue denoted cold stress. Asterisks were used to indicate a significant difference in expression compared to the control value (* *P <*0.05; * * *P <*0.01).

To further determine whether the level of *GhBRX* gene family expression was related to abiotic stress, we investigated the expression levels of 12 *GhBRX* genes via qRT–PCR. Seedlings leaves were sampled at five different stress periods (0, 1, 3, 6, 12 and 24 h) to analyze the expression of *GhBRX* genes under different abiotic stress conditions, including salt, drought, cold and heat stress (250 mM NaCl, 15% PEG, 12°C and 42°C). Under four different stresses (PEG, NaCl, cold, and heat), all the tested genes responded to at least one stress (**Figure 6**). Following a three-hour salt stress treatment, the expression levels of *GhBRX.1*, *GhBRX.2*, *GhBRXL2.4*, *GhBRXL4.1*, and *GhBRXL4.4* increased when the stress duration was extended. *GhBRXL4.5* and *GhBRXL4.6* exhibited increasing and then decreasing trends, respectively. After 3 h of drought stress treatment, the expression of *GhBRX.1*, *GhBRX.2*, *GhBRXL2.2*, *GhBRXL2.3*, *GhBRXL2.4* and *GhBRXL4.3* first increased and then decreased, and that of *GhBRXL2.1*, *GhBRXL4.1*, *GhBRXL4.2*, *GhBRXL4.4*, *GhBRXL4.5* and *GhBRXL4.6* first decreased and then increased. *GhBRX.1*, *GhBRX.2*, *GhBRXL2.2*, *GhBRXL2.3*, *GhBRXL4.1*, *GhBRXL4.2*, *GhBRXL4.3*, *GhBRXL4.4*, *GhBRXL4.5* and *GhBRXL4.6* were subjected to high-temperature stress for 3 h, after which the expression levels increased with increasing duration of stress. Similarly, the expression of *GhBRX.1*, *GhBRX.2*, *GhBRXL4.2*, *GhBRXL4.3* and *GhBRXL4.5* significantly increased after 24 h of low-temperature stress treatment compared with that at 0 h. In summary, *GhBRX.1*, *GhBRX.2* and *GhBRXL4.3*, which are highly responsive to all four kinds of stress, were selected as stress candidate genes.

### 3.8 Knockdown of the *GhBRX.1*, *GhBRX.2*, and *GhBRXL4.3* genes reduces cotton resistance to salt and cold stress

**Figure 7:**
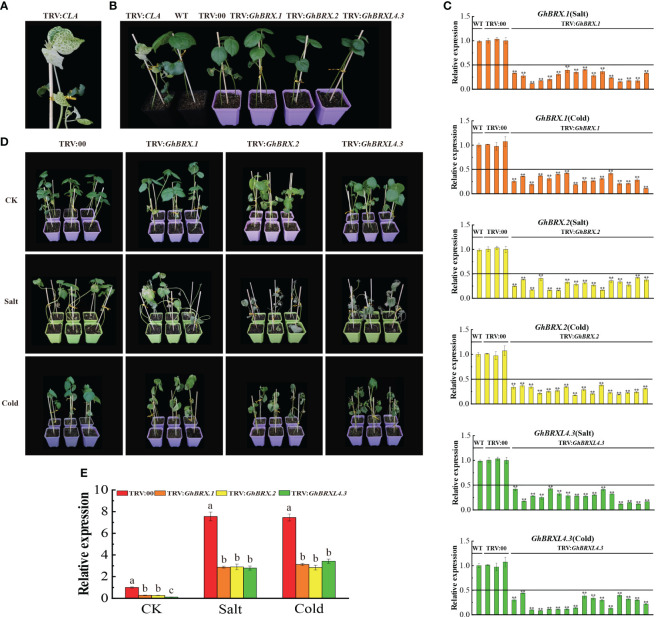
Silencing of *GhBRX.1*, *GhBRX.2* and *GhBRXL4.3* affects tolerance to salt and cold stress in upland cotton plants. **(A)** Positive control. **(B)** Representative VIGS images (TRV: *CLA*, WT, TRV: 00, TRV: *GhBRX.1*, TRV: *GhBRX.2*, and TRV: *GhBRXL4.3*). **(C)** The silencing efficiency of the WT, TRV: 00, TRV: *GhBRX.1*, TRV: *GhBRX.2* and TRV: *GhBRXL4.3* plants were tested for salt and cold stress, and the standard deviation determined from three separate experiments were represented by the error lines. **(D)** Phenotypes of the target gene-silenced plants in comparison to those of the control plants growing under normal growth conditions **(CK)** and under stress conditions (250 mmol/L NaCl, 12°C). **(E)** RT–qPCR analysis of changes in the expression levels of the *GhBRX.1*, *GhBRX.2* and *GhBRXL4.3* genes in target gene-silenced plants before and after stress treatment. “TRV: 00” represented plants carrying the empty vector control; “TRV: *GhBRX.1*, TRV: *GhBRX.2*, and TRV: *GhBRXL4.3*” represented plants with *GhBRX.1*, *GhBRX.2* and *GhBRXL4.3* silenced, respectively. The error line represented the standard deviation calculated from three independent experiments. Asterisks indicated a t test for statistically significant differences (* * *P* <0.01). Significant changes between control and gene-silenced plants were indicated by different letters (ANOVAs; *P*<0.05).

We selected the *GhBRX.1*, *GhBRX.2*, and *GhBRXL4.3* genes for further investigation based on the transcriptome and qRT–PCR results. It is assumed that the *GhBRX.1*, *GhBRX.2* and *GhBRXL4.3* genes are potentially important in the regulation of the stress response. To test our hypothesis, we used the VIGS method to knock down the *GhBRX.1*, *GhBRX.2* and *GhBRXL4.3* genes by constructing the vectors TRV: *GhBRX.1*, TRV: *GhBRX.2* and TRV: *GhBRXL4.3*, respectively. TRV: *CLA* served as a positive control (**Figures 7A, B**). Ten days after VIGS, when albino plants were observed in the positive control group, the expression levels in the leaves of the VIGS-silenced and control plants were determined via qRT–PCR. The qRT–PCR results showed that *GhBRX.1*, *GhBRX.2* and *GhBRXL4.3* were effectively repressed (**Figure 7C**). Silenced plants (TRV: *GhBRX.1*, TRV: *GhBRX.2*, TRV: *GhBRXL4.3*) and control plants (TRV: 00) were treated under different abiotic stress conditions, and the silenced plants showed more severe wilting after two weeks of salt and cold stress treatment (**Figure 7D**). Compared with those in control plants (TRV: 00), the expression levels of *GhBRX.1*, *GhBRX.2* and *GhBRXL4.3* were significantly lower after 0 days (CK) and 8 days (salt and low temperature) in the silenced plants (TRV: *GhBRX.1*, TRV: *GhBRX.2*, TRV: *GhBRXL4.3*) (**Figure 7E**). Taken together, these findings showed that cotton tolerance to cold and salt stress was decreased by silencing *GhBRX.1*, *GhBRX.2*, and *GhBRXL4.3*.

### 3.9 Physiological and biochemical indices of *GhBRX.1-*, *GhBRX.2-*, and *GhBRXL4.3*-silenced plants under salt and cold stress

**Figure 8:**
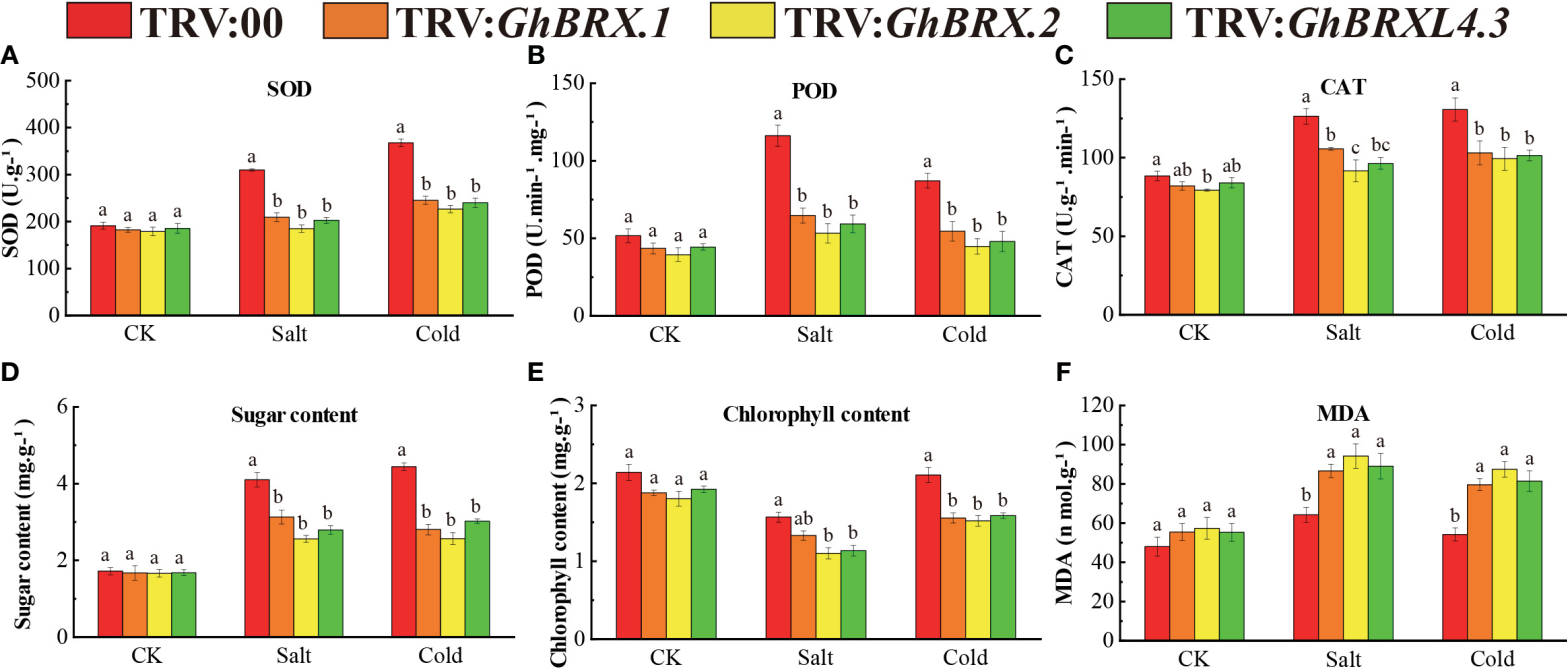
Determination of antioxidant enzyme activities and soluble sugar, chlorophyll and MDA concentrations in *GhBRX.1-*, *GhBRX.2-* and *GhBRXL4.3*-silenced plants and control plants under abiotic stress: **(A)** SOD activity; **(B)** POD activity; **(C)** CAT activity; **(D)** soluble sugar content; **(E)** chlorophyll activity; **(F)** MDA concentration. The standard deviation of three biological replicates were shown by error bars. Significant changes between control and gene-silenced plants were indicated by different letters (ANOVA; *P* < 0.05).

Plants with silenced target genes were less resistant to salt and cold stress. To investigate the impact of salt and cold stress on the silenced plants, we determined the activities of the reactive oxygen species (ROS) scavenger enzymes SOD, POD, and CAT and the MDA, soluble sugar and chlorophyll contents in the leaves before and 8 days after salt or cold stress. Under normal growth conditions, there was no significant difference in physiological parameters between the silenced plants (TRV: *GhBRX.1*, TRV: *GhBRX.2*, TRV: *GhBRXL4.3*) and the control plants (TRV:00). After 8 days of salt and cold stress, the SOD, POD and CAT activities of the silenced plants (TRV: *GhBRX.1*, TRV: *GhBRX.2*, and TRV: *GhBRXL4.3*) were significantly lower than those of the control plants (TRV: 00) (**Figures 8A–C**), indicating that the VIGS-silenced plants suffered extensive oxidative damage. The contents of soluble sugars and chlorophyll in the silenced plants were significantly lower than those in the control plants, while the content of MDA in the silenced plants was significantly greater than that in the control plants (**Figures 8D–F**), indicating that the resistance of the silenced plants decreased under adverse conditions and that the degree of adverse damage increased. The results showed that the silenced plants were very sensitive to salt stress and cold stress and that silencing the *GhBRX.1*, *GhBRX.2* and *GhBRXL4.3* genes significantly reduced their ability to tolerate salt stress and cold stress.

### 3.10 Expression analysis of stress-responsive genes in control and targeted gene-silenced plants under salt and cold conditions

**Figure 9:**
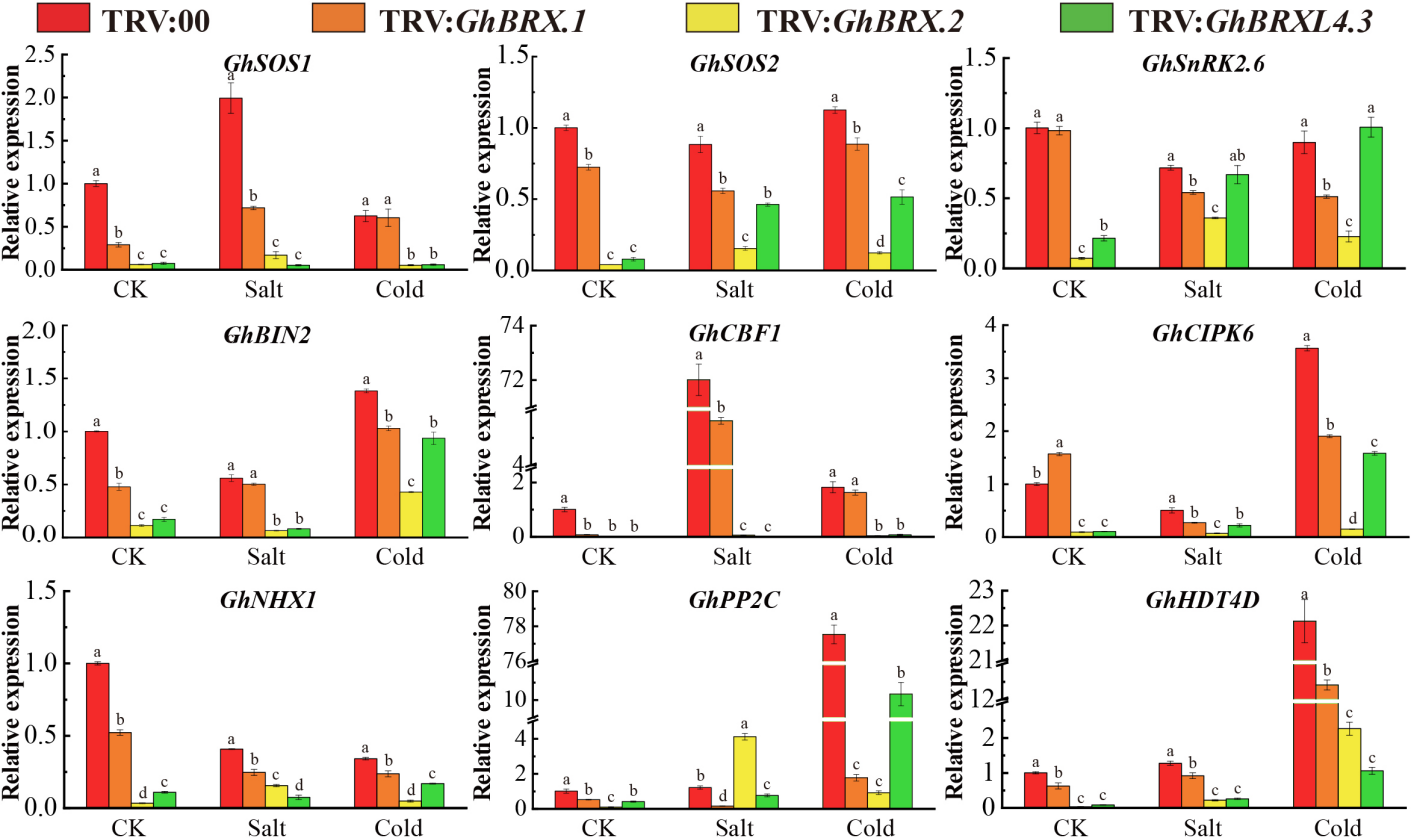
Expression of stress-responsive genes associated with salt stress tolerance or cold stress tolerance in control and silenced plants before and after stress. The error line represents the standard deviation calculated from three independent experiments. Significant changes between control and gene-silenced plants were indicated by different letters (ANOVA; *P* < 0.05).

The nine genes associated with tolerance to salt stress or low-temperature stress were selected for analysis of the response characteristics of the control and targeted gene-silenced plants under salt and cold conditions. The nine genes included *GhSOS1* (Na^+^/H^+^ antiporter salt overly sensitive 1), *GhSOS2* (salt overly sensitive 2), *GhNHX1* (Na^+^/H^+^ antiporter), *GhCIPK6* (Ser/Thr protein kinase 6), *GhBIN2* (glycogen synthase kinase 3 (GSK3)-like kinase), *GhSnRK2.6* (SnRK2 protein kinase), *GhHDT4D* (a member of the HD2 subfamily of histone deacetylases), *GhCBF1* (C-repeat binding factor) and *GhPP2C* (protein phosphatase 2C). The expression levels of these nine genes were high in the leaves of the control plants but were significantly lower in the targeted gene-silenced plants under salt or cold stress (**Figure 9**). The downregulated expression of these genes indicated that the plants were very sensitive to salt and cold stress and had a significantly reduced ability to tolerate various abiotic stress factors, resulting in greater oxidative damage.

## 4 Discussion

Drought, high temperature, salinity, and cold are all environmental stressors that harm plant growth and output (Suzuki et al., 2014), change the internal balance within plants, and affect all biological and physiological activities within plants (Aasamaa and Sõber, 2011). Therefore, it is crucial to develop new varieties with enhanced performance and maintain and improve cotton production (Kuppu et al., 2013; Kirungu et al., 2019; Hajihashemi et al., 2020; Li et al., 2021). *BRX* is involved in the control of plant embryonic development, root and bud growth, tiller angle and stomatal development (Scacchi et al., 2009; Beuchat et al., 2010b; Liu et al., 2010; Li et al., 2019; Rowe et al., 2019; Zhang Y. et al., 2021; Tiwari et al., 2023). Further studies have shown that *BRX* regulates crosstalk between signaling pathways of various phytohormones, including BRs, auxin, ABA and cytokinin (Mouchel et al., 2006; Li et al., 2009; Rodrigues et al., 2009). The *BRX* gene family in rice may be implicated in BR and auxin signaling, and *BRX* genes respond differently to abiotic stress and may play a role in the abiotic stress response (Liu et al., 2010). *TaBRXL1* is involved primarily in developmental processes, whereas *TaBRXL2* is extensively regulated by hormones, development, and other abiotic stressors (Tiwari et al., 2023).

Although *BRX* genes have been found throughout the genomes of many plant species, only rice (Liu et al., 2010) and wheat (Tiwari et al., 2023) have been found to contain evidence of their possible roles in stress adaptation. The *BRX* gene has not been identified in cotton, and its function in cotton has rarely been studied. By using the AtBRX protein as a query, we identified 12, 6 and 6 *BRX* gene family members in *G. hirsutum*, *G. raimondii* and *G. arboreum*, respectively. There are 5 *BRX* genes in *Arabidopsis* and rice, 10 *in B. rapa*, and 13 in *T. aestivum* (Briggs et al., 2006; Liu et al., 2010; Zhang Y. et al., 2021; Tiwari et al., 2023). The monocots wheat and rice clustered together with stronger homology. Among the dicotyledonous plants, the three cotton species exhibited greater similarity and formed a cluster. The allotetraploid cotton species *G. hirsutum* is the product of the doubling of the ancestral cross between two diploid cotton species, *G. raimondii* and *G. arboreum*, according to a phylogenetic tree study. Research has demonstrated that sequence similarities across genes belonging to the same taxonomic category can result in similar activities (Nan et al., 2021). The gene structure of the *GhBRX* genes is largely conserved between orthologous genes. Homologs of *GhBRX*, *GhBRX2* and *GhBRXL4* are predicted to have exons similar to those reported for *AtBRX*, *AtBRXL2* and *AtBRXL4*. The GhBRX proteins have similar MEME motifs. A few motifs have varying copy numbers in different proteins, and some motifs share two or more proteins, which could account for the functional discrepancies described among BRX family proteins.

The primary mechanism of gene family expansion is gene duplication. Segment and tandem repeats are thought to be the primary drivers of gene family expansion in plants (Cannon et al., 2004; Flagel and Wendel, 2009). Twenty segmentally duplicated genes and one tandemly duplicated gene were identified in the *GhBRX* gene family. Therefore, our study points to segmental duplication as the primary cause of the increase in *GhBRX*s. One technique for researching gene evolution and relationships is to analyze the collinearity of various species (Yu et al., 2020). The results of intergenomic symbiosis analysis between upland cotton and the other two cotton species were compared to acquire better knowledge of the homologous gene functions and evolutionary linkages of the genes. The results indicate that *BRX* gene duplication events and chromosomal rearrangements may be conserved in cotton given the numbers of *G. raimondii* and *G. arboreum*. Analysis revealed the same number of direct homologous gene pairs between *G. hirsutum* and *G. raimondii* and *G. arboreum*, indicating high conservation of *BRX* genes in cotton. To study the differences after gene duplication, the *Ka* and *Ks* values of the replicated *GhBRX* genes in upland cotton were calculated. The results showed that the *Ka/Ks* ratio of all the duplicate *GhBRX* gene pairs was <1, indicating that the *GhBRX* gene family experienced selection pressure during evolution.

Identification and characterization of *cis*-regulatory DNA sequences in response to coordinated developmental and environmental cues are critical for plant biology (Schmitz et al., 2022). We isolated the upstream promoter segments of the candidate genes and examined the distribution of *cis*-acting elements in the promoter region of *GhBRX* to gain additional insight into the potential role of *GhBRX* in upland cotton under various environmental conditions. In the present study, 38 *cis*-acting elements (those involved in stress responsiveness, tissue specificity, plant hormone responsiveness, and photoresponsiveness) were confirmed within the promoters of the *GhBRX*s. Photoresponsive elements are widely found in plants; for example, AT-rich G-boxes, GT1, Box 4, and I-boxes are commonly present in photoinducible promoters (Lam and Chua, 1989; Gilmartin et al., 1992; Foster et al., 1994). The upland cotton genes *GhBRX.1*, *GhBRX.2*, and *GhBRXL4.3* were highly expressed in roots, and *GhBRXL4.3* was also strongly expressed in pistils. These findings were based on the prediction of *cis*-elements in *GhBRX* genes and RNAseq expression data. The *GhBRX* gene promoter contains *cis*-acting regions linked to the abiotic stress response, including MBS (drought-induced MYB junction) and LTR (*cis*-acting element of cold responsiveness), suggesting that the regulation of drought and cold stress in cotton may be mediated by *GhBRX* genes. The presence of *cis*-elements in the promoter regions of genes provides clues to the spatiotemporal and hormonal regulation of genes and their response to different environmental stresses. We studied the expression of *GhBRX*s in various cotton leaves under normal and abiotic stress conditions to better understand the use of *GhBRX*s in cotton growth and abiotic stress resistance. The transcriptomic data of *GhBRX* genes in leaves under abiotic stress were obtained from the RNA-seq data of Zhejiang University, and most of the *GhBRX* genes responded to at least one stressor. In addition, to confirm the prior transcriptome findings, *GhBRX* transcript levels under abiotic stress were evaluated using quantitative RT–PCR. Quantitative RT–PCR analysis revealed that the expression of the *GhBRX.1*, *GhBRX.2* and *GhBRXL4.3* genes was significantly upregulated 24 h after the four stress treatments. *GhBRXL2.2*, *GhBRXL2.3* and *GhBRXL4.1* were significantly elevated under salt and drought stress, while *GhBRXL4.2* was significantly elevated under both heat and cold stress. These findings suggested that these genes may play a significant biological role in enhancing upland cotton tolerance to abiotic stress.

Since genes were significantly upregulated under all four stress treatments, to further explore the role of the *GhBRX.1*, *GhBRX.2* and *GhBRXL4.3* genes in abiotic stress regulation in upland cotton, we constructed a VIGS vector for further functional analysis. We treated the silenced plants with abiotic stress (salt, drought, high temperature and low temperature) and found that the silenced plants were more sensitive to salt stress and low-temperature stress, and the silenced plants exhibited a more obvious phenotype and water loss phenotype. These findings may indicate that the *GhBRX.1*, *GhBRX.2* and *GhBRXL4.3* genes may play significant biological roles in enhancing tolerance to salt and cold stress in cotton plants. To investigate the stress-related mechanisms of these three genes, we analyzed the physiological and biochemical indicators, including ROS scavenger enzyme (SOD, POD, CAT) activities and MDA, soluble sugar and chlorophyll contents, of silenced plants and control plants before and after stress. The induction of salt stress and cold stress leads to the overproduction of ROS and other oxygen radicals, leading to oxidative destruction of plant cell structure and their components and eventually plant death; antioxidant defense systems work together to control uncontrolled oxidative cascades and protect plant cells from oxidative damage by removing ROS (Gill and Tuteja, 2010; Malhan et al., 2015). Therefore, the removal of excess ROS is a key process for plant protection against salt stress and cold stress (Zhang et al., 2011; Ullah et al., 2018). Essential ROS scavenging enzymes include SOD, POD, and CAT, whose activities increase in plants exposed to cold and salt stress (Salih et al. 2024). The results showed that SOD, POD, and CAT enzyme activities increased significantly after stress due to the overproduction of plant ROS in upland cotton, and the ability to eliminate ROS was significantly reduced after the silencing of *GhBRX.1*, *GhBRX.2* and *GhBRXL4.3*. Therefore, after stress, the SOD, POD and CAT activities of the *GhBRX.1-*, *GhBRX.2-* and *GhBRXL4.3*-silenced plants significantly decreased compared with those of the control plants. The MDA concentration is a crucial indicator of the body’s ability to respond to antioxidants and can also be used to infer the extent of cell damage (Yu et al., 2016). The soluble sugar content can reflect not only the growth status of crops but also their quality (Jiang et al., 2020). After stress, the degree of oxidative damage to the cotton plants increased, the MDA content of the silenced plants increased, and the soluble sugar and chlorophyll contents decreased, indicating that the VIGS-mediated silencing of these plants increased cell damage and decreased quality. This further revealed the important role of the proteins encoded by the *GhBRX.1*, *GhBRX.2* and *GhBRXL4.3* genes in enhancing salt tolerance and low-temperature tolerance in cotton.

Finally, we examined the expression levels of nine known stress response genes, *GhSOS1*, *GhSOS2*, *GhNHX1*, *GhCIPK6*, *GhBIN2*, *GhSnRK2.6*, *GhHDT4D*, *GhCBF1* and *GhPP2C*, in the leaf tissue of VIGS and control plants (TRV:00) under salt and cold stress conditions. Under salt and cold stress conditions, most stress-related genes exhibited considerable downregulation. The *SOS1* and *SOS2* genes can improve the salt tolerance of transgenic plants (Liu et al., 2000; Yue et al., 2012). Before and after salt stress, the expression of both *GhSOS1* and *GhSOS2*, including *GhBRX.2* and *GhBRXL4.3*, was downregulated, and the two gene-silenced plants were the most significantly downregulated. The *SnRK2.6* and *CBF1* genes play important roles in improving salt and cold tolerance in plants under stress (Novillo et al., 2007; Song et al., 2016; Wang et al., 2018; Yan et al., 2023). *PCaP2* can activate the *CBF* and *SnRK2* transcriptional networks and play an important role in cold stress tolerance (Wang et al., 2018). When subjected to salt and cold stress, although *GhSnRK2.6* was not significantly expressed in the *GhBRXL4.3* gene-silenced plants, both *GhSnRK2.6* and *GhCBF1* were downregulated in the VIGS-treated plants. *BIN2* interacts with and phosphorylates the *CBF EXPRESSION1* inducer (*ICE1*) to inhibit *SOS2* kinase activity and further inhibit the salt stress response, thus negatively regulating salt stress and low-temperature stress (Ye et al., 2019). Similarly, compared with that in the absence of stress, *GhBIN2* expression in silenced plants was broadly upregulated. The *CIPK6*, *NHX1*, *PP2C* and *HDT4D* genes play important roles in salt stress tolerance and cold stress and can be used to regulate growth and development and improve crop tolerance to salt and low-temperature stress (Teakle et al., 2010; Chen et al., 2013; Dubrovina et al., 2015; Imran et al., 2020; Zhang Y. et al., 2021; Zhu et al., 2022; Wu et al., 2023). Compared with those in control plants, except for in *GhBRX.2* gene-silenced plants, the expression of the *CIPK6*, *NHX1*, *PP2C* and *HDT4D* genes was significantly lower in VIGS-treated plants. The expression of stress-related genes in VIGS–generated cotton leaves revealed that silencing *GhBRX.1*, *GhBRX.2*, and *GhBRXL4.3* affected the expression of genes involved in the cotton stress response under salt and cold stress conditions, suggesting that *BRX* genes play an important role in upland cotton tolerance to salt and cold stress.

## 5 Conclusion

In summary, the cotton genome encodes 24 highly conserved *BRX* genes. The *BRX* genes in upland cotton have similar gene structures. Multiple *cis*-acting regions linked with hormonal or abiotic stress responses can be found in the *GhBRX* promoter sequence. qRT–PCR data also showed that different abiotic stresses could induce *GhBRX* expression. Further functional characterization of *GhBRX.1*, *GhBRX.2* and *GhBRXL4.3* by VIGS indicated that silencing of the *GhBRX.1*, *GhBRX.2* and *GhBRXL4.3* genes may weaken the response of cotton to salt and low-temperature stress. This work could lead to additional research on the function of *GhBRX*s in the cotton response and resistance to abiotic stress.

## Data availability statement

The datasets presented in this study can be found in online repositories. The names of the repository/repositories and accession number(s) can be found in the article/**Supplementary Material.**

## Author contributions

WW: Writing – original draft, Writing – review & editing, Data curation, Formal Analysis, Investigation, Validation, Visualization, Conceptualization, Methodology, Project administration, Software, Supervision. JJ: Validation, Writing – review & editing, Software. XuZ: Writing – review & editing. PL: Writing – review & editing, Validation. JL: Writing – review & editing, Validation. YL: Writing – review & editing, Validation. WX: Writing – review & editing, Validation. JS: Writing – review & editing, Funding acquisition, Resources, Supervision. XiZ: Writing – review & editing, Funding acquisition, Resources, Supervision. CW: Writing – review & editing, Formal Analysis, Funding acquisition, Resources, Supervision.
