# Supplementary material for: GhBRX.1, GhBRX.2, and GhBRX4.3 improve resistance to salt and cold stress in upland cotton
Source: Front Plant Sci. 2024 Feb 9;15:1353365. doi: 10.3389/fpls.2024.1353365 (PMC10884310; doi:10.3389/fpls.2024.1353365)
Supplement: Supplementary file 1 [file DataSheet_1.docx]

Supplementary Material

# Supplementary Tables and Figures

## Supplementary Tables

Table S1 Primer pair sequences for qRT‒PCR analysis.

| **Gene** | **Sense primer (5'–3')** | **Antisense primer (5'–3')** |
| --- | --- | --- |
| *GhBRX.1* | GCCCAAAGAATGGATGGCAC | AACATCTCCCGGCTGAATCG |
| *GhBRX.2* | CCCAAGGAATGGATGGCACA | AACATCTCCCGGCTGAATCG |
| *GhBRXL2.1* | GACTCAGCACGCTTCCATTG | TACGGCCACTAACGGAAACC |
| *GhBRXL2.2* | ACCTAGGACTAGGCAAGCCA | GAGTCGGCATAGTTCCGGTT |
| *GhBRXL2.3* | CATCCGTTAGTGGCCGTACA | CTCCTTCAGGCAGTGAGACG |
| *GhBRXL2.4* | AACTACGCAGACTCCGATGC | TCTCTTTGCCCCAACTTCCG |
| *GhBRXL4.1* | GAAGCCGACTCGGATCGTTA | TCAACCTCGCTTCCATCTCC |
| *GhBRXL4.2* | TTGAGGAACATGGGCGAGTC | GCTCGAACTCGACATCCCTT |
| *GhBRXL4.3* | GGTGTACAGGCATTGCAACC | TCCTGCGATACGACCACTTG |
| *GhBRXL4.4* | GCACTACCCACACCACCTAT | ACACTCATCCCTGTTGGACG |
| *GhBRXL4.5* | AAGGGATGTCGAGTTGGAGC | GGTAATAAGCACGCCAGGCT |
| *GhBRXL4.6* | TCCAATCGGTCGTGTTCGTC | ACCGTATCCGCTTGAGATCG |
| *GhActin* | ATCCTCCGTCTTGACCTTG | TGTCCGTCAGGCAACTCAT |

Table S2 VIGS vector primer construction.

| **Gene** | **Sense primer (5'–3')** | **Antisense primer (5'–3')** |
| --- | --- | --- |
| *GhBRX.1* | GCCCAAAGAATGGATGGCAC | CTCGCATTGCTCACCGAAAC |
| *GhBRX.2* | TGAGCCCAAGGAATGGATGG | CTCGCATTGCTCACCGAAAC |
| *GhBRXL4.3* | TTCCAGAAGAATAATGCTGACGTG | TGGCTAACCTCATTTGATTTGGTC |
| TRV: *GhBRX.1* | CCGGAATTCGCCCAAAGAATGGATGGCAC | CGACGCGTCTCGCATTGCTCACCGAAAC |
| TRV: *GhBRX.2* | CCGGAATTCTGAGCCCAAGGAATGGATGG | CCGCTCGAGCTCGCATTGCTCACCGAAAC |
| TRV: *GhBRXL4.3* | CCGGAATTCAGGTCTGAAGATGAGAGCTC | CGGGGTACCCTCTTCCCACCACAGTCTAG |

Table S3 Fluorescence quantification primers related to stress response genes.

| **Gene** | **Sense primer (5'–3')** | **Antisense primer (5'–3')** |
| --- | --- | --- |
| *GhSOS1* | AGTGTCAGCCAATAAACAACC | TCTTTCGTGTCCATCTTCTTC |
| *GhSOS2* | TATGATGGTGCTGTGGCAGACG | CCCCATAGGAAACCAAGAAGGGC |
| *GhSnrk2.6* | CACGGTTGGAACTCCTGCAT | TAACCCCGCATGACCAAACA |
| *GhBIN2* | TCTACCCCTGTTGTTGACCG | TGTCCCAACTACACGCTCTG |
| *GhCBF1* | TCGTGTTACCCCAAGAAGCG | TCCCGAGCCAAATCCTTGAC |
| *GhCIPK6* | GCCAAAGGTCGTCTCAAGGA | CAGAAATCGACGGCGGAAAC |
| *GhNHX1* | TTCTCTTTCTTTATGTCGGGATG | AACAAGACCCATCAGCACAGC |
| *GhPP2C* | TTTGTGTCGAAATGGTGCCG | TTACTTTGCCACCGGCTTCT |
| *GhHDT4D* | GAAGCCAGAAAGGCATCGGCTA | TGCAAGGCCATTTCAGAGCCAA |

Table S4 Detailed information on the *BRX* gene family members in *Gossypium hirsutum* (*Gh*), *Gossypium raimondii* (*Gr*) and *Gossypium arboreum* (*Ga*).

| **Gene name** | **Gene ID** | **Protein Lengh(aa）** | **MW（kDa）** | **pI** | **Chr** | **Chr location** | **subcellular location** |
| --- | --- | --- | --- | --- | --- | --- | --- |
| *GhBRX.1* | *GH_A12G2229* | 342 | 38.61 | 5.73 | A12 | 100620830-100623966 | nucleus |
| *GhBRX.2* | *GH_D12G2247* | 342 | 38.58 | 5.73 | D12 | 54905379-54908523 | nucleus |
| *GhBRXL2.1* | *GH_A05G1391* | 368 | 41.15 | 7.12 | A05 | 12781859-12785124 | nucleus |
| *GhBRXL2.2* | *GH_A05G4266* | 366 | 41.15 | 6.98 | A05 | 109543524-109547341 | nucleus |
| *GhBRXL2.3* | *GH_D04G0110* | 366 | 41.15 | 6.98 | D04 | 1388080-1391847 | nucleus |
| *GhBRXL2.4* | *GH_D05G1406* | 368 | 41.23 | 7.1 | D05 | 11708479-11711708 | nucleus |
| *GhBRXL4.1* | *GH_A05G0325* | 370 | 41.72 | 8.54 | A05 | 3177168-3181054 | nucleus |
| *GhBRXL4.2* | *GH_A06G0818* | 370 | 42.1 | 8.59 | A06 | 18661658-18665705 | nucleus |
| *GhBRXL4.3* | *GH_A13G2210* | 372 | 41.98 | 6.44 | A13 | 105927072-105939499 | nucleus |
| *GhBRXL4.4* | *GH_D05G0333* | 370 | 41.71 | 8.54 | D05 | 2755366-2759306 | nucleus |
| *GhBRXL4.5* | *GH_D06G0798* | 370 | 42.15 | 8.22 | D06 | 13585871-13589893 | nucleus |
| *GhBRXL4.6* | *GH_D13G2194* | 372 | 42.02 | 7.07 | D13 | 60017269-60022630 | nucleus |
| *GrBRX* | *Gorai.008G220800* | 342 | 38.58 | 5.73 | Chr08 | 50733292-50738766 | nucleus |
| *GrBRXL2.1* | *Gorai.009G143900* | 371 | 41.01 | 7.06 | Chr09 | 10893100-10898488 | nucleus |
| *GrBRXL2.2* | *Gorai.012G011200* | 366 | 41.14 | 6.98 | Chr12 | 1267709-1272293 | nucleus |
| *GrBRXL4.1* | *Gorai.009G034500* | 405 | 45.59 | 7.09 | Chr09 | 2588592-2592881 | nucleus |
| *GrBRXL4.2* | *Gorai.010G086500* | 370 | 42.19 | 7.67 | Chr10 | 13061334-13066413 | nucleus |
| *GrBRXL4.3* | *Gorai.013G226100* | 372 | 42.08 | 6.7 | Chr13 | 54539383-54545713 | nucleus |
| *GaBRX* | *Ga12G0771* | 342 | 38.58 | 5.73 | Chr12 | 7213117-7216251 | nucleus |
| *GaBRXL2.1* | *Ga04G2032* | 355 | 40.1 | 8.15 | Chr04 | 97212253-97216087 | nucleus |
| *GaBRXL2.2* | *Ga05G1462* | 368 | 41.16 | 7.65 | Chr05 | 12948949-12952217 | nucleus |
| *GaBRXL4.1* | *Ga05G0345* | 370 | 41.75 | 8.54 | Chr05 | 3154294-3158187 | nucleus |
| *GaBRXL4.2* | *Ga06G0792* | 370 | 42.14 | 8.59 | Chr06 | 14522676-14526723 | nucleus |
| *GaBRXL4.3* | *Ga13G2392* | 372 | 41.98 | 6.44 | Chr13 | 119171889-119177485 | nucleus |

Note: A total of 24 cotton *BRX* genes were identified, and their gene IDs, gene names, protein length (aa), molecular weight (MW), isoelectric point (pI), chromosomal position and subcellular localization were listed.

Table S5 Segmental duplication and tandem duplication gene pairs among *GhBRX* family members.

| **No.** | **Segmental duplication gene pairs** | **Tandem duplication gene pairs** |
| --- | --- | --- |
| 1 | *GhBRX.1*/*GhBRX.2* | *GhBRXL2.1*/*GhBRXL2.2* |
| 2 | *GhBRXL2.1*/GhBRXL2.3 |  |
| 3 | GhBRXL2.1/*GhBRXL2.4* |  |
| 4 | *GhBRXL2.2*/*GhBRXL2.3* |  |
| 5 | *GhBRXL2.2*/*GhBRXL2.4* |  |
| 6 | *GhBRXL2.3*/*GhBRXL2.4* |  |
| 7 | *GhBRXL4.1*/*GhBRXL4.2* |  |
| 8 | *GhBRXL4.1*/*GhBRXL4.4* |  |
| 9 | *GhBRXL4.1*/*GhBRXL4.5* |  |
| 10 | *GhBRXL4.1*/*GhBRXL4.6* |  |
| 11 | *GhBRXL4.2*/*GhBRXL4.3* |  |
| 12 | *GhBRXL4.2*/*GhBRXL4.4* |  |
| 13 | *GhBRXL4.2*/*GhBRXL4.5* |  |
| 14 | *GhBRXL4.2*/*GhBRXL4.6* |  |
| 15 | *GhBRXL4.3*/*GhBRXL4.4* |  |
| 16 | *GhBRXL4.3*/*GhBRXL4.5* |  |
| 17 | *GhBRXL4.3*/*GhBRXL4.6* |  |
| 18 | *GhBRXL4.4*/*GhBRXL4.5* |  |
| 19 | *GhBRXL4.4*/*GhBRXL4.6* |  |
| 20 | *GhBRXL4.5*/*GhBRXL4.6* |  |

Table S6 *Ka* and *Ks* values of homologous pairings.

| **Gene ID** | **Gene ID** | ***Ka*** | ***Ks*** | ***Ka*/*Ks*** |
| --- | --- | --- | --- | --- |
| *GH_A05G1391.1* | *GH_A05G4266.1* | 0.071244199 | 0.430764931 | 0.16538997 |
| *GH_A05G0325.1* | *GH_A06G0818.1* | 0.06944078 | 0.453792828 | 0.153023088 |
| *GH_A05G1391.1* | *GH_D04G0110.1* | 0.067327911 | 0.396729123 | 0.169707509 |
| *GH_A05G4266.1* | *GH_D04G0110.1* | 0.005976723 | 0.031656529 | 0.188799048 |
| *GH_A05G0325.1* | *GH_D05G0333.1* | 0.001170504 | 0.011857955 | 0.09871041 |
| *GH_A05G1391.1* | *GH_D05G1406.1* | 0.00830869 | 0.031813915 | 0.261165293 |
| *GH_A05G4266.1* | *GH_D05G1406.1* | 0.069890483 | 0.424982762 | 0.164454865 |
| *GH_A05G0325.1* | *GH_D06G0798.1* | 0.070686013 | 0.440444325 | 0.160487964 |
| *GH_A05G0325.1* | *GH_D13G2194.1* | 0.080092442 | 0.516917282 | 0.154942473 |
| *GH_A06G0818.1* | *GH_A13G2210.1* | 0.061947182 | 0.385269638 | 0.16078916 |
| *GH_A06G0818.1* | *GH_D05G0333.1* | 0.068189457 | 0.445788878 | 0.152963567 |
| *GH_A06G0818.1* | *GH_D06G0798.1* | 0.005843996 | 0.03255852 | 0.179492055 |
| *GH_A06G0818.1* | *GH_D13G2194.1* | 0.060086713 | 0.374432495 | 0.160474089 |
| *GH_A12G2229.1* | *GH_D12G2247.1* | 0.003787887 | 0.044391654 | 0.085328807 |
| *GH_A13G2210.1* | *GH_D05G0333.1* | 0.080075982 | 0.525289827 | 0.152441524 |
| *GH_A13G2210.1* | *GH_D06G0798.1* | 0.062548873 | 0.363200764 | 0.172215697 |
| *GH_A13G2210.1* | *GH_D13G2194.1* | 0.010528541 | 0.052760084 | 0.199555048 |
| *GH_D04G0110.1* | *GH_D05G1406.1* | 0.065983429 | 0.391139809 | 0.168695253 |
| *GH_D05G0333.1* | *GH_D06G0798.1* | 0.069433705 | 0.432585198 | 0.16050874 |
| *GH_D05G0333.1* | *GH_D13G2194.1* | 0.078825556 | 0.492865183 | 0.159933302 |
| *GH_D06G0798.1* | *GH_D13G2194.1* | 0.060688167 | 0.352700899 | 0.172066947 |

Note: non-synonymous substitutions (*Ka*) and synonymous substitutions (*Ks*).

## 1.2 Supplementary Figures
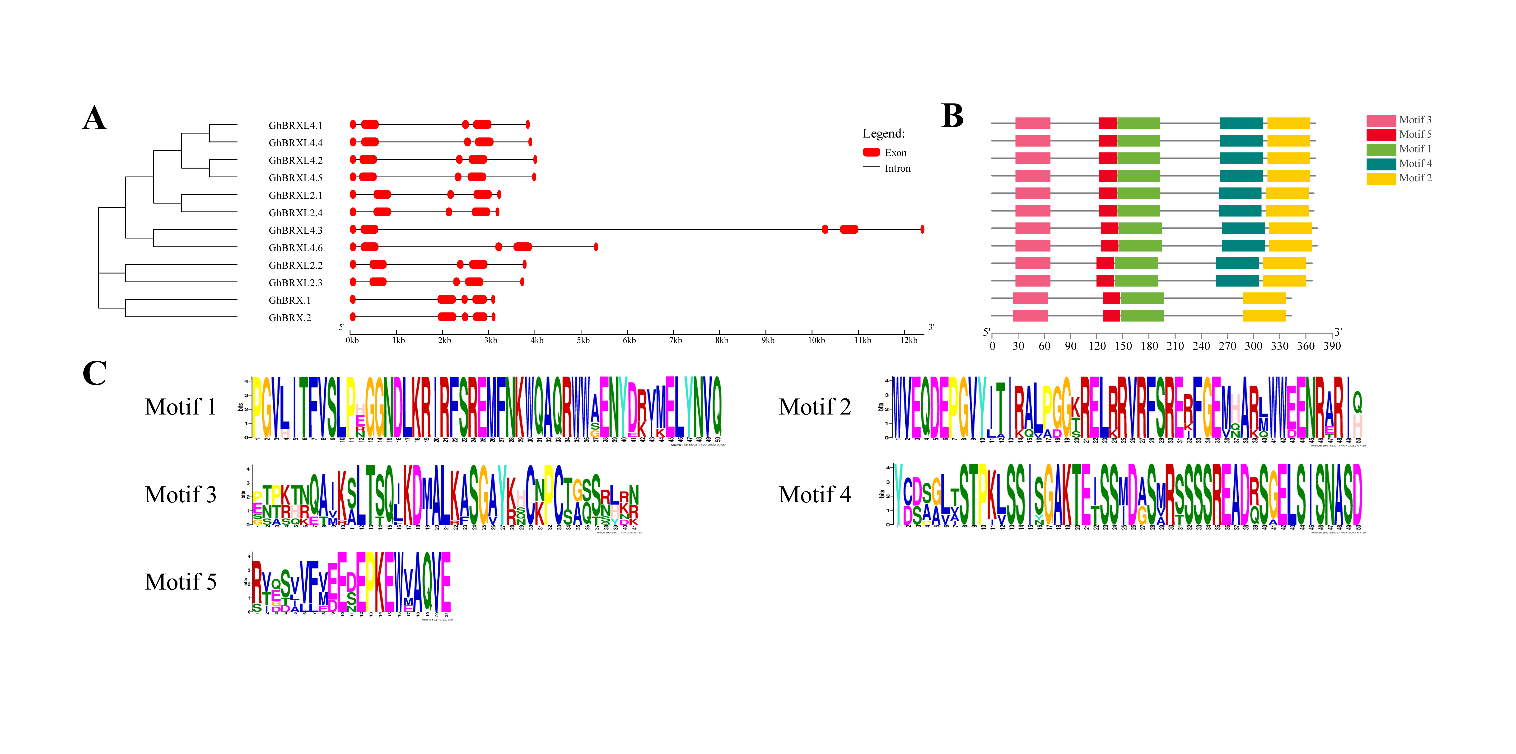


**Figure S1** Structures, motif compositions and domains of *GhBRX* genes. **(A)** *GhBRX* exon/intron structure and unrooted phylogenetic tree. **(B)** Conserved motifs of 12 GhBRX proteins. **(C)** Conserved motif analysis of the sequence logo.


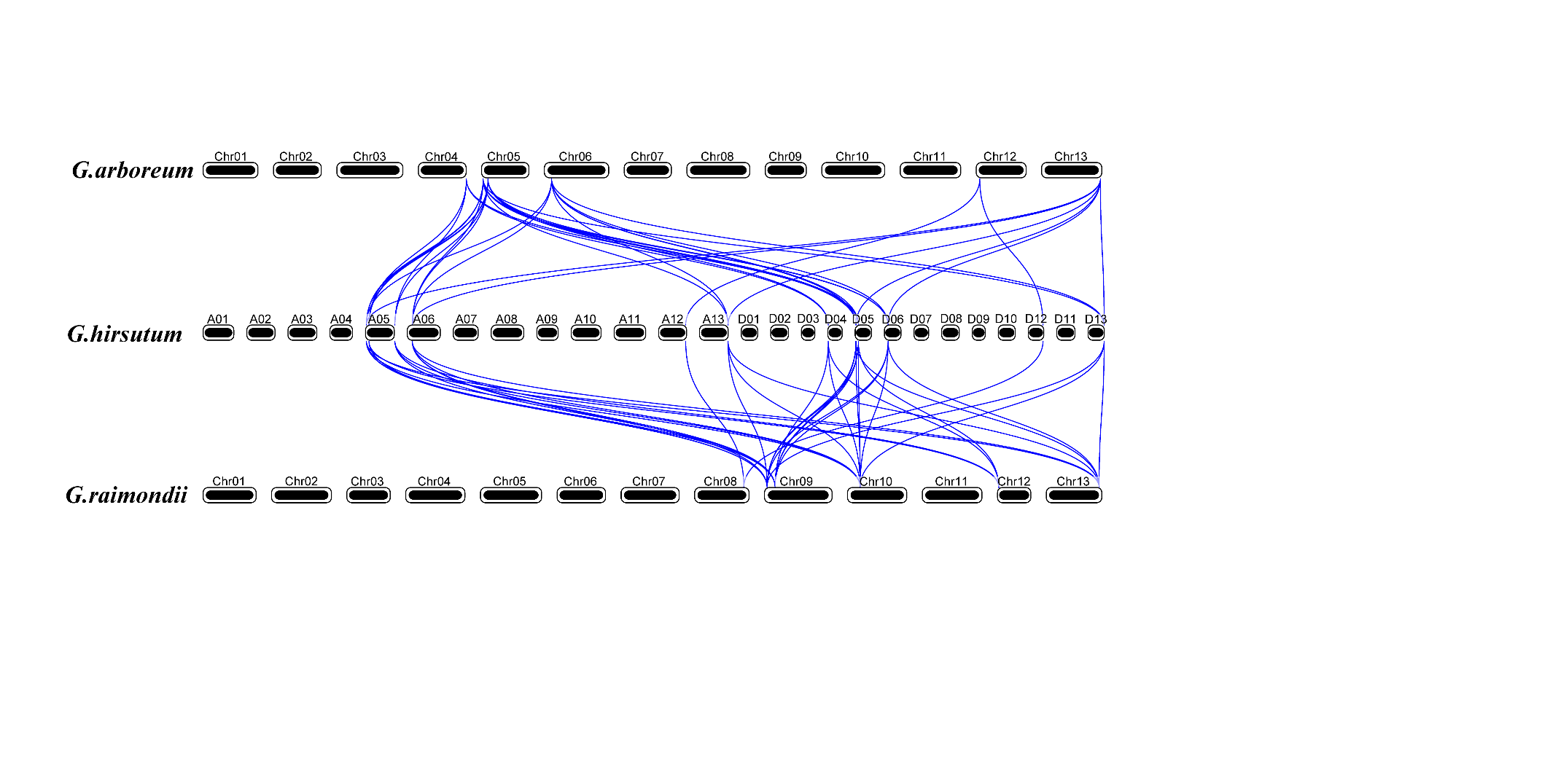
 **Figure S2** Synteny analysis of *BRX* genes between *G. hirsutum* and *G. raimondii* and *G. arboreum*. The blue lines indicated the syntenic *BRX* gene pairs.
